# Maternal uniparental isodisomy for chromosome 6 discovered by paternity testing: a case report

**DOI:** 10.1186/s13039-018-0411-3

**Published:** 2018-12-20

**Authors:** Elizabeth R. Kerr, Gary M. Stuhlmiller, George C. Maha, Mark A. Ladd, Fady M. Mikhail, Ruth P. Koester, Anna C. E. Hurst

**Affiliations:** 1 School of Medicine, University of Alabama at Birmingham, 1670 University Blvd, Birmingham, AL 35233 USA; 2 DNA Identification Testing Division, Laboratory Corporation of America Holdings, 1440 York Court, Burlington, NC 27215 USA; 3 Department of Genetics, University of Alabama at Birmingham, 720 20th St. S, Birmingham, AL 35294 USA

**Keywords:** Maternal uniparental disomy, Chromosome 6, Paternity testing, Isodisomy

## Abstract

**Background:**

Uniparental disomy (UPD) is a rare condition in which a child inherits both copies of a chromosome or chromosome segment from one parent. Medical consequences of UPD may include abnormal imprinting, unmasking of genetic disease, and somatic mosaicism; alternatively, the condition may be clinically silent. We present a case of maternal UPD for chromosome 6, a rare condition previously reported less than 20 times. In our patient with a normal phenotype, the condition was discovered through abnormal paternity testing results. Uniparental isodisomy is a rare cause of discordant parentage testing results, but it is an important phenomenon to recognize.

**Case presentation:**

We present a female born at 32 weeks gestational age with birth weight 10–25%ile when corrected for prematurity. Paternity testing was obtained for legal reasons, and initial results appeared to exclude the alleged father. However, the lab performed additional testing which indicated that the patient was homozygous for maternal alleles for all three tested loci located on chromosome 6. Based on these results, the patient was referred for a medical genetics evaluation for possible maternal uniparental disomy. She presented for her consultation at 10 months of age and appeared to be developing appropriately. Her age-adjusted weight, length, and head circumference were <3%ile, 10%ile, and 25%ile respectively. Chromosomal microarray testing confirmed maternal UPD6. The patient was seen again at 14 months of age, and her weight and length were 10–25%ile. She had not developed concerning symptoms or physical exam findings.

**Conclusions:**

The presence of UPD, especially in asymptomatic patients, has implications for paternity testing, as standard methods may miss cases of both isodisomy and heterodisomy. This rare inheritance pattern should be considered when discordant paternity results come under suspicion. It is unusual for a parentage testing lab to perform the amount of testing done for this case, but the initial inconsistencies necessitated further investigation. UPD6 has uncertain effects and variable phenotypes, so this patient’s genetic abnormality likely would have gone undiscovered if not for the non-medical indication for the laboratory analysis. Her asymptomatic presentation raises the possibility that UPD may be more common than previously estimated.

## Background

Uniparental disomy (UPD) is a rare condition, first described in 1980, in which a child inherits both copies of a chromosome or chromosome segment from one parent [1]. The estimated incidence is 1 in 3500 live births [2]. In isodisomy, identical copies of one chromosome homologue (i.e., sister chromatids) are inherited from one parent. It is due to a non-disjunction error in meiosis II followed by trisomy rescue or a monosomy conception followed by mitotic monosomy rescue [3]. In heterodisomy, two different homologues of the same chromosome (i.e., homologous chromosomes) are inherited from one parent due to a non-disjunction error in meiosis I followed by trisomy rescue [3].

The medical consequences of UPD may include abnormal imprinting (seen in Angelman and Prader-Willi syndromes) or unmasking of autosomal recessive disease; additionally, mosaic segmental UPD is the genetic mechanism for a portion of Beckwith-Wiedemann syndrome cases [3]. UPD may be suspected based on the presence of a characteristic phenotype or other clinical suspicion prompting genetic evaluation. However, prior reports have described discovery of UPD through paternity testing done for legal reasons involving children who had no clinical features that would warrant medical genetics testing [4–8].

Maternal UPD6 is extremely rare with less than 20 cases reported [9]. Intrauterine growth restriction (IUGR) appears to be the most common phenotype observed in affected patients; no clear pattern is observed among the other reported findings [10]. In contrast, paternal UPD6 has an associated phenotype of transient neonatal diabetes, and it is estimated to be the cause of about 50% of sporadic cases of this disease [11].

This case report describes a female patient with a nonspecific medical history who was found to have maternal uniparental isodisomy for chromosome 6 when her family questioned the results of a routine paternity test performed for legal reasons. Our aim is to add to the literature on UPD6 by highlighting this patient’s rare genetic condition and normal phenotype in the setting of the unconventional manner in which it was discovered. Uniparental isodisomy is a rare cause of discordant maternity or paternity testing results, but it is an important phenomenon to recognize for those performing such testing.

## Case presentation

We present a female born via Caesarean section at 32 weeks gestational age to a 31-year-old gravida 4 para 3 (now para 4) mother. The pregnancy course was uncomplicated, and no prenatal genetic testing was indicated. Delivery was emergent due to fetal heart rate decelerations, and after delivery a nuchal cord was noted. Birth weight was 1304 g (10–25%ile for gestational age). The patient stayed in the neonatal intensive care unit for 2 months, during which her course was complicated by intraventricular hemorrhage of unknown grade.

Paternity testing was obtained a few months after birth due to court regulations involving the patient’s parents. Samples from the patient, mother, and alleged father were analyzed using PowerPlex© 16 and CS7 in accordance with standard laboratory practices. A total of 21 polymorphic loci were genotyped. Initial results appeared to exclude the alleged father from paternity due to genetic inconsistencies at loci F13A01 and D5S818. However, the mother insisted on the alleged father’s paternity, and additional testing was subsequently performed. As part of the process for resolution of this unique case, the lab tested PowerPlex© ESX, PowerPlex© Fusion, and PowerPlex© LC5 test batteries. HLA testing was performed as well. Of note was the finding that the patient was homozygous for maternal alleles for all loci located on chromosome 6 (see Table 1). These findings prompted the laboratory to recommend that the patient receive a medical genetics evaluation for possible maternal uniparental disomy. Table 1 summarizes the genetic irregularities associated with chromosome 6 that led to suspicion of the underlying condition.

**Table 1:** Chromosome 6 genetic testing results suggested the possibility of uniparental disomy

Tested person	HLA	F13A01	D6S1043
Mother	A33, A-; B7, B53	6, 14	11, 13
Child	A33, A-; B7, B-	6	11
Alleged father	A23, A33; B35, B58	7	12, 18

The patient presented for a medical genetics consultation at 10 months of age following the updated test results. During the visit, her mother reported that the patient appeared to be developing well and reaching milestones appropriately. No concerning symptoms were discovered on review of systems. Upon physical examination, her weight was less than the 3rd percentile (even when corrected for prematurity) and length was less than the 3rd percentile (10th percentile when corrected for prematurity). Head circumference was at the 25th percentile. The exam was otherwise unremarkable except for small preauricular pits. Her family history was notable for a maternal half-brother with attention-deficit/ hyperactivity disorder and grandparents with hypertension.

**Fig. 1:**
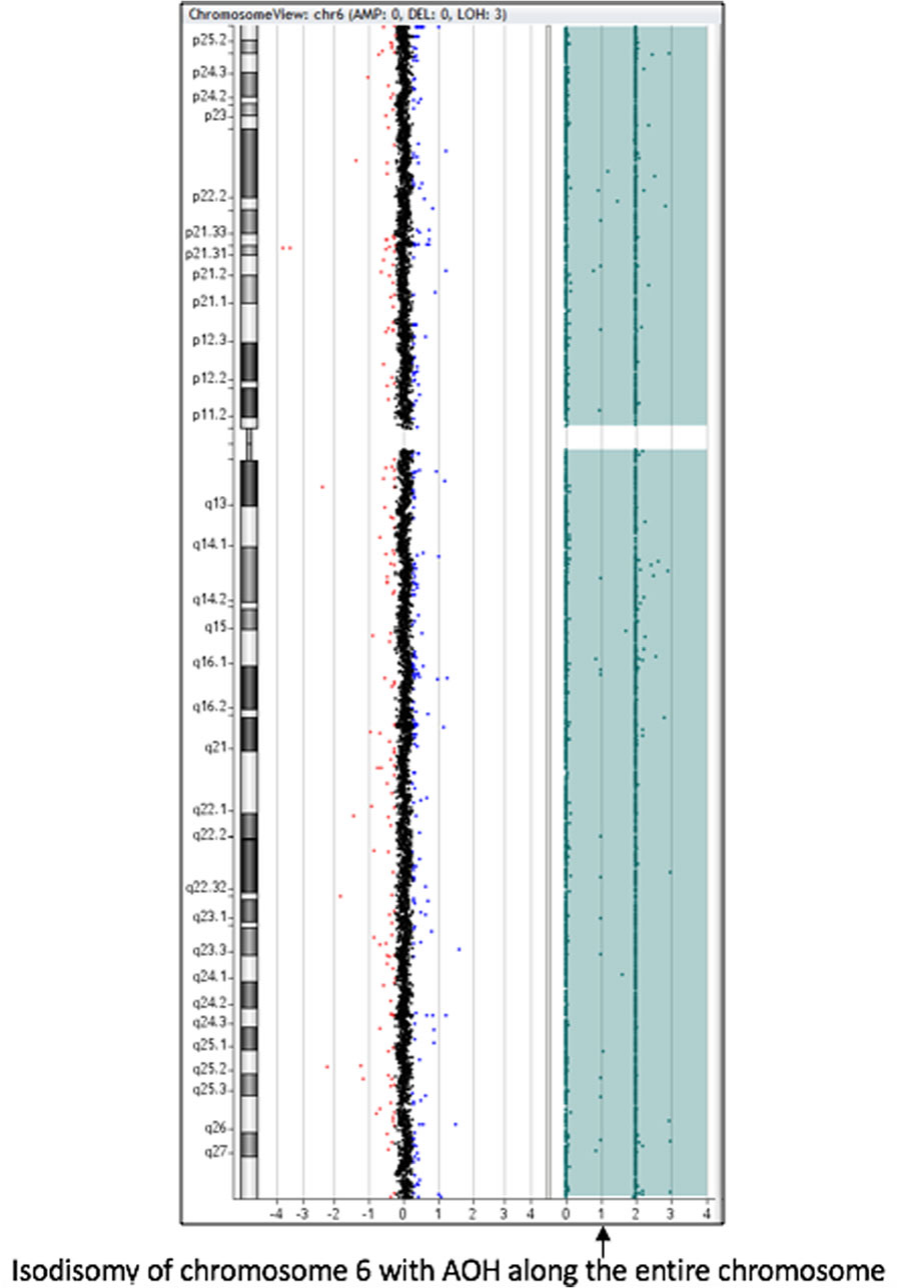
Results from the CMA analysis revealed two extended contiguous regions of homozygosity spanning and entire short and long arms of chromosome 6. AOH = absence of heterozygosity

Due to potential health implications of UPD, chromosomal microarray (CMA) testing was ordered to confirm chromosomal composition. CMA analysis using Agilent 4x180k aCGH+SNP array supported the diagnosis of maternal UPD6 (Fig. 1). Of note, after confirming this diagnosis, the additional inconsistency on chromosome 5 was concluded to be an unrelated single inconsistency. A single inconsistency in paternity testing is usually interpreted as an inconsequential mutation, and for the D5S818 locus these single inconsistencies are seen in about 0.17% of paternity cases [12].

The patient was next seen at 14 months of age to discuss the results of the CMA. At this time, her weight and length were at the 5th percentile (10–25th percentile when corrected for prematurity). She had continued to reach developmental milestones and had no new symptoms or concerning physical exam findings. The family was counseled on the possibility of the patient developing an autosomal recessive condition due to unmasking caused by the UPD. Overall, however, her prognosis is good based on her reassuring first two visits.

## Discussion and conclusions

UPD is a condition in which a child inherits both homologues of a chromosome from one parent. In isodisomy, the individual inherits two identical copies of one homologue from one parent, whereas in heterodisomy, the individual inherits two different homologues from one parent. Our patient had isodisomy, which was ultimately detected and displayed by the genetic testing results, including CMA aCGH+SNP testing. Heterodisomy, on the other hand, could by missed CMA SNP testing due to the heterozygosity of the uniparental homologs; however, most cases with heterodisomy present with a mixture of hetero- and isodisomy due to crossing over, which would allow their detection by CMA SNP testing.

The presence of UPD, especially in asymptomatic patients, has implications for paternity testing results, as the standard routine screening methods have the potential to miss cases of both types of this condition. Even a more extensive workup utilizing a CMA SNP array testing can miss cases of uniparental pure heterodisomy. This inheritance pattern, although extremely rare, should be considered when discordant paternity results come under suspicion. If all of the genetic inconsistencies noted are at loci on the same chromosome, further testing is warranted.

It is highly unusual for a parentage testing lab to perform the amount of testing that was done for this case. The lab pursued further testing because of the genetic inconsistencies between the child and the alleged father at D5S818 and F13A01 found in its initial testing approach. Two inconsistencies could have represented actual exclusions or genetic variants, so this necessitated further testing. The HLA results provided the third inconsistency. Finally, with the result for D6S1043, the lab realized the uniqueness of the findings at chromosome 6. HLA testing is a last resort used only for the most challenging of cases. F13A01 is part of an extended test battery not used for standard parentage testing. Given this patient’s eventual UPD diagnosis, the D5 results were consistent with single paternal inconsistency, which is seen in an approximate frequency of 0.0017 in the general population [12].

UPD can have medical consequences caused by imprinting problems, unmasking of recessive disease, and somatic mosaicism. Some forms of UPD are well-known and well-studied, such as Prader-Willi, Angelman, and Beckwith-Wiedemann syndromes. Others have uncertain effects and variable phenotypes, such as maternal UPD for chromosome 6. The patient we describe has some notable aspects of her medical history, such as premature birth at 32 weeks gestational age and corrected weight less than the 3rd percentile at 10 months of age. Her weight had normalized by her 14-month visit. In comparison, the main associated phenotype for maternal UPD6 reported in the literature is IUGR, which was observed in 8 of 9 reported isodisomy cases [10]. Several of these patients were noted to have catch-up growth or normal postnatal growth. Other phenotypic findings are varied in nature and severity and do not form a consistent clinical profile.

Because of this patient’s nonspecific medical history and lack of significant anomalies, her genetic abnormality likely would not have been discovered if not for the non-medical indication for the laboratory analysis. UPD may be more common than previously estimated, as expanded genetic testing is rarely indicated in asymptomatic patients.

## Abbreviations

aCGH: array comparative genomic hybridization
CMA: Chromosomal microarray
IUGR: Intrauterine growth restriction
SNP: Single nucleotide polymorphism
UPD: Uniparental disomy
